# The utility of using peak expiratory flow and forced vital capacity to predict poor expiratory cough flow in children with neuromuscular disorders

**DOI:** 10.4102/sajp.v75i1.1296

**Published:** 2019-06-27

**Authors:** Brenda M. Morrow, Lauren Angelil, Juliet Forsyth, Ashleigh Huisamen, Erin Juries, Lieselotte Corten

**Affiliations:** 1 Department of Paediatrics and Child Health, University of Cape Town, Cape Town, South Africa; 2 Division of Physiotherapy, Department of Health and Rehabilitation Sciences, University of Cape Town, Cape Town, South Africa

**Keywords:** neuromuscular disorders, peak cough flow, forced vital capacity, spirometry, paediatric

## Abstract

**Background:**

Approximately one in every 1200 South Africans is affected by a neuromuscular disease (NMD). Weak respiratory muscles and ineffective cough contribute to the development of respiratory morbidity and mortality. Early identification of individuals at risk of respiratory complications, through peak expiratory cough flow (PCF) measurement, may improve patient outcomes through timely initiation of cough augmentation therapy.

**Objectives:**

The aim of this study was to investigate the relationship between peak expiratory flow (PEF), forced vital capacity (FVC) and PCF in South African children with neuromuscular disorders.

**Methods:**

A retrospective descriptive study of routinely collected data was conducted.

**Results:**

Forty-one participants (aged 11.5 ± 3.6 years; 75.6% male) were included. There was a strong linear correlation between PCF and PEF (*R* = 0.78; *p* = 0.0001) and between PCF and FVC (*R* = 0.61; *p* = 0.0001). There was good agreement between PCF and PEF, with intraclass correlation coefficient of 0.8 (95% confidence interval, 0.7–0.9; *p* < 0.0001). Peak expiratory flow < 160 L.min^−1^ and FVC < 1.2 L were significantly predictive of PCF < 160 L.min^−1^ (suggestive of cough ineffectiveness), whilst PEF < 250 L.min^−1^ was predictive of PCF < 270 L.min^−1^, the level at which cough assistance is usually implemented.

**Conclusion:**

PEF and FVC may be surrogate measures of cough effectiveness in children with neuromuscular disorders.

**Clinical implications:**

PEF and FVC may be considered for clinical use as screening tools to identify patients at risk for pulmonary morbidity related to ineffective cough.

## Introduction

Children with neuromuscular disorders (NMD) typically present with mild to severe muscle weakness that may extend to the respiratory muscles, depending on the condition and stage of the disease. Although children with NMD initially have normal lungs and mucociliary clearance mechanisms, advancing respiratory muscle weakness leads to progressive pulmonary impairment and reductions in total lung capacity. Respiratory muscle weakness also contributes to rib cage deformity and, together with reduced mobility and potentially poor sitting posture, may result in chest wall deformities, which further impacts respiratory function (Panitch 2009:S215–S218). Shortening and fibrosis of the chest wall muscles, because of an inability to fully expand the chest as a consequence of inspiratory muscle weakness, result in a progressive decrease in chest wall compliance, leading to an eventual restrictive pattern of respiratory disease (Gozal 2000:141–150; Panitch 2009:S215–S218).

The inability to cough effectively, owing to respiratory muscle weakness, predisposes the patient to secretion retention, which leads to airway obstruction, increased work of breathing, hypoxia and potentially respiratory failure (Chatwin et al. 2018:98–110). Depending on the NMD condition, all components and co-ordination of the cough may be affected, including an inability to take a sufficiently deep inspiration; lack of glottic closure; poor expiratory muscle length–tension relationship; reduced chest wall recoil and insufficient expiratory muscle strength necessary for an expulsive exhalation (Bach et al. 2006:105–111; Boitano 2006:913–922; Finder 2010:31–34; Hull et al. 2012:i1–40; Toussaint et al. 2009:359–366). In the long term, together with chronic low-volume breathing and loss of sigh capacity, there is likely to be further loss of lung compliance, recurrent infections and potentially chronic lung disease (Boitano 2006:913–922; Panitch 2009:S215–S218; Sharma 2009:S219–21; Toussaint et al. 2009:359–366). Most reported cases of respiratory failure in NMD are related to ineffective coughing during intercurrent chest infections (Bach et al. 2006:105–111). Furthermore, most causes of death in people with NMD are because of respiratory failure and pneumonia; therefore, prevention and early treatment are of paramount importance in their management (Boitano 2006:913–922; Dohna-Schwake et al. 2006:325–328).

Early identification of children who require cough assistance could prevent morbidity and delay mortality by instituting airway clearance regimens as part of the child’s home programme. By regularly clearing secretions from the lungs, the consequences of secretion retention could be minimised, potentially leading to fewer hospitalisations for the management of intercurrent infections and reducing the need for mechanical ventilatory support (Chatwin et al. 2018:98–110; Hull et al. 2012:i1–40; Toussaint et al. 2018:289–298).

Peak cough flow (PCF) and other pulmonary function tests are commonly measured in clinical practice globally, to assess cough effectiveness and document progression of pulmonary impairment in adults and children with NMD (Chatwin et al. 2018:98–110). Peak cough flow is the peak expiratory flow measured during a cough; however, in contrast with other pulmonary function measures, there are, as yet, no standardised testing guidelines (Chatwin et al. 2018:98–110), and this test is not commonly used in the South African setting, despite international recommendations (Toussaint et al. 2018:289–298). In children with NMD, aged 4–20 years, PCF has been shown to be a good predictor of the risk of developing acute respiratory complications, with PCF < 160 L.min^−1^ predictive of severe disease and the level below which a cough is likely to be ineffective in adults (Bach, Ishikawa & Kim 1997:1024–1028; Bianchi & Baiardi 2008:461–467; Dohna-Schwake et al. 2006:325–328; Hull et al. 2012:i1–40; Toussaint et al. 2018: 289–298). A study of patients with Duchenne Muscular Dystrophy (DMD) suggested that PCF should be above 270 L.min^−1^ when healthy, to account for the predictable decline in PCF during intercurrent chest infections (Bach et al. 1997:1024–1028), and that cough augmentation treatment should be implemented when PCF falls to below this level in adults and children over 12 years of age (Toussaint et al. 2018: 289–298). This pre-morbid PCF cut-off has not been tested in younger children. Normal, age-adjusted PCF values have been reported for children, which reach the lower limit of adult values from 12 years of age (Bianchi & Baiardi 2008:461–467). Below age 12, the critical values of PCF required for an effective cough are not yet clear.

The correlation between PCF and other variables (such as vital capacity [VC], maximal inspiratory capacity, total lung capacity, expiratory reserve volume, maximal inspiratory pressure and maximal expiratory pressure) has been investigated in adults with NMD (Trebbia et al. 2005:291–300). Forced vital capacity (FVC) and peak expiratory flow rate (PEF) have been shown to be well correlated in 10–18-year-olds with DMD (Meier et al. 2017:307–314). Similarly, Bach et al. (2006:105–111) reported significant correlation between PCF and both PEF and VC in patients with a range of NMDs, but paediatric and adult results were not reported separately. Gauld and Boynton (2005) also evaluated the relationship between spirometry and PCF in children with DMD, and reported an association between both forced expiratory volume in 1 s (FEV_1_) and FVC, and PCF, with identified values of both FEV_1_ and FVC predictive of poor PCF (Gauld & Boynton 2005:457–460). Studies on patients with DMD may not be generalisable to other NMDs, owing to the known impact of the condition on cognition and the inherent gender bias.

Lung function assessments can be lengthy and may be difficult to perform with young children owing to co-operation and comprehension challenges. In addition, not all measurement modalities may be available, particularly in low-resourced environments, including many regions within South Africa. It is important to ensure that routine tests are both efficient and effective, to minimise healthcare resource utilisation and patient fatigue, whilst optimising patient participation and co-operation. If there were significant agreement between standard spirometry measures and PCF, there may be no need to perform all measurements for every child at every clinic visit, thereby potentially saving time and resources and reducing patient fatigue. Toussaint et al. recently provided recommendations for cough assistance in people with NMD, using adult and older adolescent values of FVC and PCF to determine the level and type of cough support needed (Toussaint et al. 2018:289–298). To make similar recommendations for younger children with a range of NMD, it is first necessary to establish the relationship between PCF and FVC.

This study aimed to describe the level of agreement and correlation between measures of PEF, FVC and PCF in South African children with NMD. We also aimed to identify specific PEF and FVC values to predict the threshold levels of PCF, which are generally associated with ineffective cough and respiratory morbidity.

## Materials and methods

This was a retrospective descriptive folder review of routinely collected clinical data. Children aged 5 to 18 years with any diagnosed NMD, attending the NMD outpatient clinic at Red Cross War Memorial Children’s Hospital in Cape Town, South Africa, were eligible for inclusion if their records were available for review and they had performed an acceptable quality lung function test (as per American Thoracic Society [ATS] criteria), with a paired PCF and PEF or VC measurement, between January 2012 and July 2017. There were no exclusion criteria.

During the study period, unassisted PCF had been measured routinely during clinic visits using a hand-held Wright’s Peak Flow Meter and mouthpiece interface, and results recorded in the patient’s clinical notes for each clinic visit. Peak expiratory flow and FVC were measured using a MicroLoop^TM^ Pulmonary Function device (CareFusion, Germany), according to ATS criteria (‘Standardization of Spirometry, 1994 Update. American Thoracic Society’ 1995:1107–1136). Peak cough flow measures and clinical and demographic data were extracted from participants’ medical folders, whilst the date-matched spirometry measures of FVC and PEF were downloaded from the MicroLoop^TM^ device. The MicroLoop^TM^ automatically indicates in the report whether pulmonary function results were acceptable according to ATS requirements.

### Statistical analysis

Continuous data were tested for normality using the Shapiro–Wilk’s *W* test. Descriptive statistics are presented as median (range or interquartile range [IQR]) if non-normal; mean ± standard deviation (SD) if normally distributed or n (%) as appropriate. We evaluated the probability–probability (P–P) plots for PCF, PEF and FVC to determine variance. Spearman’s rank order and Pearson’s correlation tests were used along with scatter plots with best linear fit and regression algorithms to determine the relationships between PCF and PEF or FVC, and to identify cut-off values of PEF and FVC equivalent to two critical levels of PCF (< 160 L.min^−1^ and < 270 L.min^−1^, respectively). Binary logistic regression was conducted to determine the odds ratios of these values of PEF and FVC to predict the critical PCF levels. Level of agreement between PEF and PCF was assessed using a Bland–Altman plot and by calculating the intraclass correlation coefficient (ICC).

Intraclass correlation coefficient estimates and 95% confidence intervals (CIs) were calculated based on a single measures, absolute agreement, two-way mixed-effects model. The effects of FVC, PCF and PEF on ambulation status, steroid use and the presence of a chest wall deformity (recorded at the same visit as the lung function measurements) were analysed using the Mann–Whitney *U* or *t* test for independent variables (according to distribution). The significance level was set at 5%, and the Bland–Altman limits of agreement are expressed as 1.96 × SD of the mean difference. IBM SPSS Statistics version 24 (IBM Corporation) and Statistica version 13 (StatSoft Inc., Tulsa, OK, USA) were used for statistical analysis.

### Ethical considerations

Institutional Human Research Ethics Committee approval (Ref: 205/2017) was obtained for the study, and telephonic consent for enrolment was obtained from the parents or legal guardians of the children.

## Results

**FIGURE 1:**
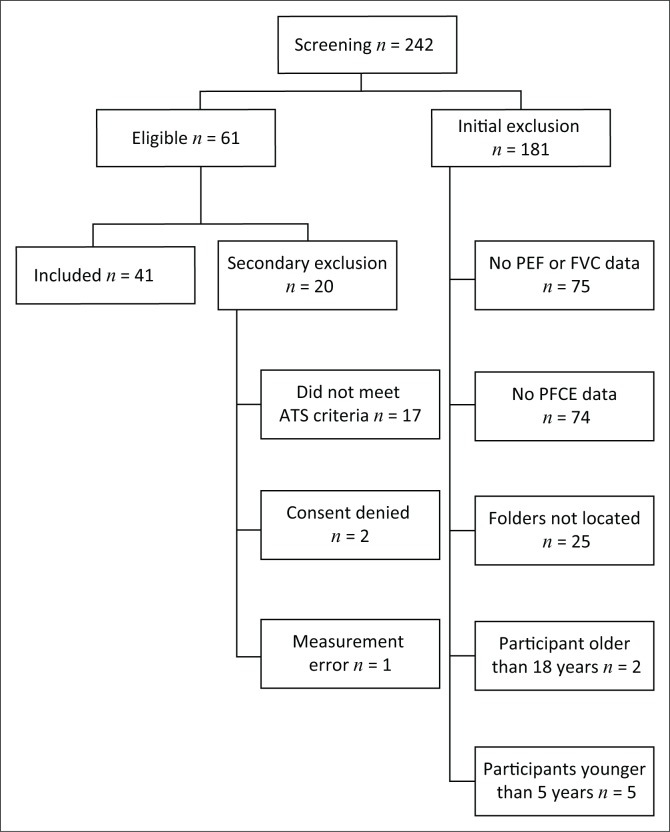
Flowchart of participants through the study.

Of the 242 patients with NMD who were assessed for eligibility, 41 participants (aged 11.5 ± 3.6 years) were included in the final sample for analysis (Figure 1). The majority of participants (75.6%) were male, and DMD was the most common NMD condition (43.9%) (Table 1). Eighteen (43.9%) participants were not ambulant; two (4.9%) had respiratory exacerbations at the time of pulmonary function testing and two (4.9%) received nocturnal non-invasive ventilatory support (Table 1). Eleven (26.8%) participants had PCF < 160 L.min^−1^ and pulmonary function was generally poor, with a mean FVC *Z* score of less than −3 (Table 1).

**TABLE 1:** Participant characteristics (*n* = 41).

Variable	Data
Male gender	31 (75.6)
Age at spirometry test (years) (mean ± SD)	11.5 ± 3.6
Age at diagnosis (years) (mean ± SD)	5.1 ± 3.2
Body mass index (*n* = 35) (median [IQR])	16.5 (13.2–20.7)
Underweight (< 5th percentile)	11 (31)
Normal BMI (5th–85th percentile)	11 (31)
Overweight or obese (≥ 85th percentile)	13 (37)
Obese (≥ 95th percentile)	5 (14)
NMD condition	
Duchenne Muscular Dystrophy	18 (43.9)
Congenital myopathy	5 (12.2)
Charcot–Marie Tooth disease	4 (9.7)
Congenital muscular dystrophy	2 (4.9)
Limb girdle muscular dystrophy	2 (4.9)
Spinal muscular atrophy type II	3 (7.3)
Other NMD	7 (17.1)
Comorbid condition	
Asthma	1 (2.4)
Hypertension	1 (2.4)
Non-ambulant	18 (43.9)
Thoracic deformity	14 (34.1)
Scoliosis	11 (26.8)
Kyphosis	2 (4.9)
Kyphoscoliosis	1 (2.4)
Receiving systemic steroids	14 (34.1)
Nocturnal non-invasive ventilation	2 (4.9)
Number of previous spirometry measurements (median [IQR])	2 (4.5–8.0)
PCF (L.min^−1^) (mean ± SD)	220.0 ± 98.2
PEF (L.min^−1^) (median [IQR])	199.0 (155.0–253.0)
FVC (L) (median [IQR])	1.6 (1.05–1.9)
FVC (percentage predicted) (median [IQR])	55.0 (42.2–91.0)
FVC (GLI *Z* score) (mean ± SD)	−3.09 ± 2.65

NMD, neuromuscular disorder; IQR, interquartile range; SD, standard deviation; PCF, peak expiratory cough flow; PEF, peak expiratory flow; FVC, forced vital capacity; GLI, Global Lung Function Initiative. Categorical variables are presented as *n* (%) throughout.

Peak cough flow was normally distributed, but PEF and FVC were non-normal. However, the P–P plots for all measures (PCF, PEF and FVC) showed very little variance from the straight-line relationship, and it was therefore considered acceptable to analyse and plot both non-linear and linear relationships between these variables.

**FIGURE 2:**
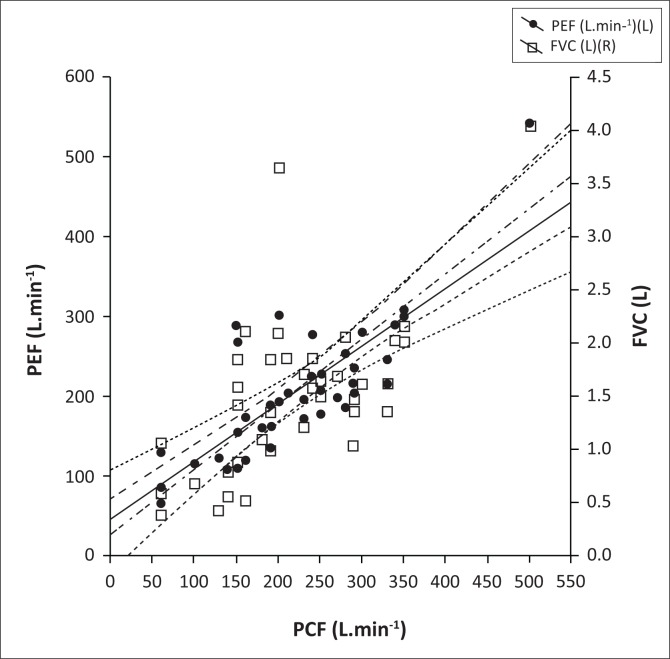
Correlation between peak cough flow (PCF) and peak expiratory flow (PEF) (Pearson’s *R* = 0.81; *r*^2^ = 0.65; *p* < 0.0001) and forced vital capacity (FVC) (Pearson *r* = 0.67; *r*^2^ = 0.44; *p* < 0.0001); *n* = 41. Linear regression equations: PEF = 26.46+0.82 ´ PCF; FVC = 0.24 + 0.005 ´ PCF.

There were highly significant correlations between PCF and both PEF (Pearson *r* = 0.81; *p* < 0.0001) and FVC (Pearson *r* = 0.67; *p* < 0.0001), with the strongest correlation between PCF and PEF (Figure 2). Using percentage predicted values of FVC, the adjusted odds ratio for predicting PCF of 160 L.min^−1^ and 270 L.min^−1^, respectively, were 0.96 (95% CI, 0.92–0.99; *p* = 0.01) and 0.98 (95% CI, 0.96–1.0; *p* = 0.1).

**FIGURE 3:**
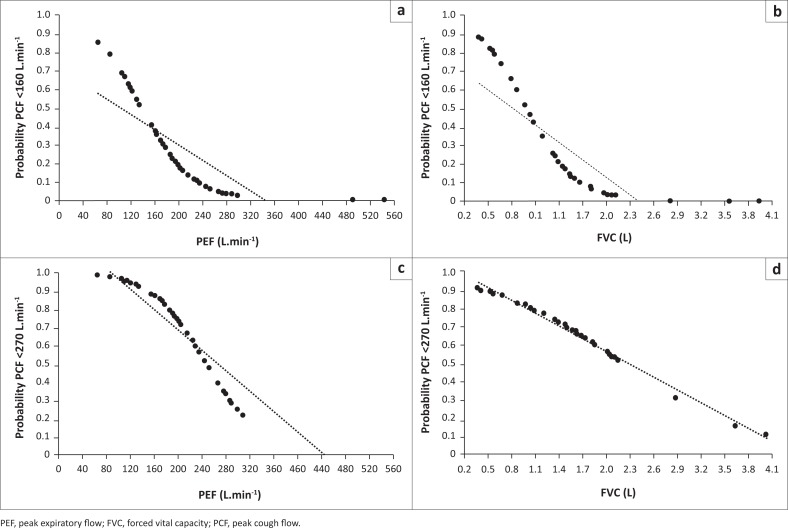
Probability plots for peak expiratory flow and forced vital capacity and peak cough flow values of (a), (b) < 160 L.min^−1^ and (c), (d) < 270 L.min^−1^.

**FIGURE 4:**
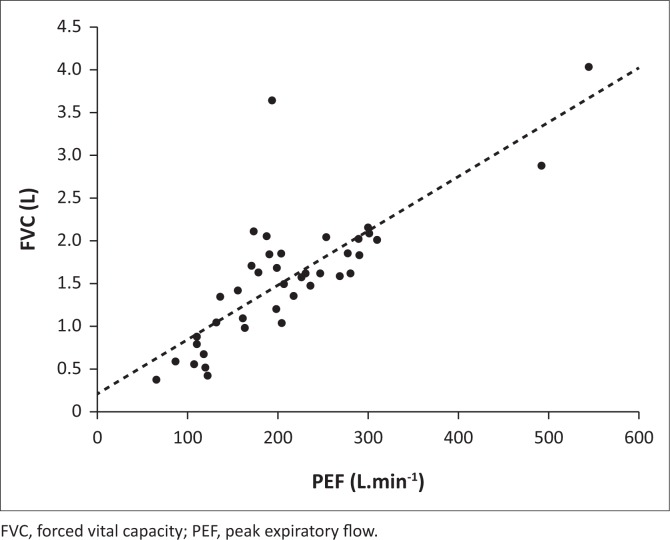
Correlation between forced vital capacity and peak expiratory flow (Pearson’s *R* = 0.79; *r*^2^ = 0.62; *p* < 0.0001); *n* = 41.

Using the linear regression equations (Figure 2), the cut-off PCF values of < 160 L.min^−1^ and < 270 L.min^−1^ corresponded with a PEF of <160 L.min^−1^ and < 250 L.min^−1^ and an FVC of < 1.2 L and < 1.8 L, respectively, which corresponded to the inflection points on the logistic regression-generated probability curves (Figure 3a–d). There was also a significant linear correlation between PEF and FVC (Figure 4).

Both the identified cut-offs of FVC and PEF were excellent predictors of PCF < 160 L.min^−1^, but the predictive value declined for PCF < 270 L.min^−1^ (Table 2).

**TABLE 2:** Odds ratios for cut-off levels of peak expiratory flow and forced vital capacity and corresponding peak expiratory cough flow thresholds.

Variable	Odds ratio	95% confidence interval	*p*
**PCF < 160 L.min**^−1^			
PEF < 160 L.min^−1^	63	7.7–513.9	0.0001
FVC < 1.2 L	17.3	3.2–94.3	0.001
**PCF < 270 L.min**^−1^			
PEF < 250 L.min^−1^	5.75	1.3–25.6	0.02
FVC < 1.8 L	2.86	0.74–11.1	0.1

PCF, peak expiratory cough flow; PEF, peak expiratory flow; FVC, forced vital capacity.

**FIGURE 5:**
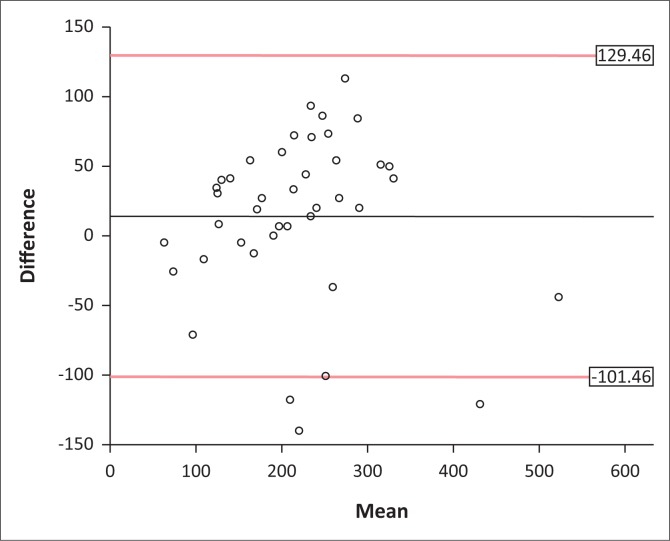
Bland–Altman plot comparing the difference between peak cough flow (PCF) and peak expiratory flow (PEF) (PCF–PEF) and mean (PCF–PEF). The upper and lower limits of agreement are set at mean difference (PCF–PEF) ± 1.96 ´ standard deviation of the differences. The central horizontal line indicates the mean difference (14.0 L.min^−1^).

Figure 5 presents the Bland–Altman plot of agreement between PCF and PEF. The plot shows an acceptable bias, with a mean difference between PCF and PEF of 14 L.min^−1^ and > 95% of the data points falling within the accepted limits of agreement, as recommended (Giavarina 2015:141–151). The ICC for PEF and PCF was 0.8 (95% CI, 0.7–0.9; *p* < 0.0001), indicating good reliability, and Cronbach’s alpha was 0.89, indicating good internal consistency.

Ambulation status and systemic steroid use had no effect on PEF, FVC or PCF (*p* ≥ 0.1). The presence of a chest wall deformity was associated with significantly lower PEF (*p* = 0.048), with no effect on PCF or FVC (Table 3).

**TABLE 3:** Comparison of participant characteristics and pulmonary function measures.

Variable	Chest wall deformity present			Ambulation status			Receiving systemic steroids		
	Yes (*n* = 14)	No (*n* = 23)	*p*	Ambulant (*n* = 22)	Non-ambulant (*n* = 18)	*p*	Yes (*n* = 14)	No (*n* = 26)	*p*
PEF (L.min^−1^)	182.5(110.0–204.0)	217.0 (161.0–277.0)	0.048	185.5 (136.0–230.0)	203.5 (161.0–277.0)	0.6	203.5 (173.0–268.0)	190.0 (119.0–253.0)	0.3
FVC (L)	1.3 (0.6–1.7)	1.6(1.35–2.0)	0.200	1.5(1.1–2.0)	1.6 (1.0–1.9)	0.9	1.7 (1.6–1.9)	1.5 (0.8–2.0)	0.1
PCF (L.min^−1^) (mean ± SD)	197.9 ± 97.7	240.4 ± 92.9	0.200	226.4 ± 95.8	215.6 ± 89.2	0.7	225.0 ± 64.7	225.0 ± 108.9	1.0

PEF, peak expiratory flow; FVC, forced vital capacity; PCF, peak expiratory cough flow; SD, standard deviation.

Data are presented as median (interquartile range) unless otherwise stated.

There were insufficient numbers to analyse subgroups of ventilated versus non-ventilated, and obese versus non-obese participants. Participant age was positively correlated with PEF (Spearman *R* = 0.50; *p* =0.0008), FVC (Spearman *R* = 0.39; *p* = 0.01) and PCF (Pearson *R* =0.4; *p* = 0.006).

## Discussion

This is the first study to report on the association between PCF, PEF and FVC in South African children with NMD. Our results show a strong correlation between PCF and both PEF and FVC, similar to that reported previously in different population groups (Bach et al. 2006:105–111), with PEF a better predictor of PCF than FVC. Performing adequate and repeatable pulmonary function tests may be lengthy and lead to patient fatigue. Our results suggest that it may not be necessary to perform both spirometry and PCF tests for all patients, at every clinic visit. This approach may reduce patient fatigue whilst optimising resource utilisation and time efficiency.

For an effective cough, one needs adequate inspiration to above 80% of total lung capacity, glottic closure and a coordinated active exhalation with maximal expiratory flows combined with expulsive glottis opening (Boitano 2006:913–922; Chatwin et al. 2018:98–110). Cough efficacy depends, therefore, on both inspiratory and expiratory capacity, and hence inspiratory and expiratory muscle strength. We therefore focused our study on measures of lung capacity (FVC) and expiratory flow measures of PEF and PCF.

Peak cough flow values lower than 160 L.min^−1^ have been shown to be a good predictor of an ineffective cough, and indicate those at risk of developing acute respiratory complications in children with NMD between the ages of 4 and 20 years (Bianchi & Baiardi 2008:461–467; Dohna-Schwake et al. 2006:325–328; Hull et al. 2012:i1–40).

The critical value of PCF of 160 L.min^−1^ corresponded with a PEF of approximately 160 L.min^−1^ and FVC of 1.2 L, values below which were highly predictive of PCF < 160 L.min^−1^. To our knowledge, this has not previously been reported in this age group. Our findings suggest that it may be possible to screen for young patients with NMD who are likely to be prone to respiratory morbidity, using either PEF or FVC, with routine PCF measurements and cough assistance techniques considered once values fall below PEF 250 L.min^−1^ and FVC 1.8 L. The actual values of PCF corresponding to clinically ineffective cough in young children under 12 years have not yet been established and warrant further investigation. Furthermore, the large number of patients who were excluded from this study on the basis of poor quality of forced spirometry, possibly owing to fatigue in those with marked respiratory muscle weakness, suggests that measures of relaxed vital capacity may be preferable to measuring FVC in these children, and this warrants further investigation.

It has been suggested that assistance with the cough is needed once PCF falls below 270 L.min^−1^, to account for the inevitable decline in cough efficacy in the event of a respiratory tract infection (Bach et al. 1997:1024–1028). Gauld and Boynton (2005) identified FVC values < 2.1 L as being highly predictive of PCF < 270 L.min^−1^ in boys with DMD (Gauld & Boynton 2005:457–460). We were unable to demonstrate a significant relationship between our identified FVC cut-off value (1.8 L) and the likelihood of PCF being less than 270 L.min^−1^. However, we did demonstrate that PEF values < 250 L.min^−1^ were predictive of PCF < 270 L.min^−1^. This has not previously been reported. Our study differs from Gauld and Boynton’s in terms of the population studied (boys with DMD in the latter study vs. heterogeneous population of children with different NMDs in our study). Similar to the previous study (Gauld & Boynton 2005:457–460), we used a mouthpiece interface to measure PCF, although a recent consensus review (Chatwin et al. 2018:98–110) recommended that a facemask be used. Gauld and Boynton (2005) reported that spirometry was considered easier to perform than PCF manoeuvres in their study population, and this warrants further investigation in children with different NMD conditions. Our findings suggest that regular PEF measurement may be useful as a surrogate marker of children at risk of pulmonary morbidity, but this requires further investigation. It is further suggested that when PEF falls below 250 L.min^−1^, regular PCF measurement be implemented, wherever possible, and airway clearance therapy with cough assistance be taught to the patient and caregivers, as per current recommendations for adolescents and adults (Toussaint et al. 2018:289–298).

There was very little difference between PCF and PEF in our study of children with a range of NMD (insignificant mean difference of 14 L.min^−1^), whereas in a previous study of adults and children with NMD, PCF values were found to be significantly greater than PEF (Bach et al. 2006:105–111). Interestingly, the difference between PCF and PEF was no longer significant in a sub-analysis of children only, similar to our findings (Bach et al. 2006:105–111). Suarez et al. (2002) reported that PCF was greater than PEF in their sub-cohort of children with DMD, with a significant PCF–PEF mean difference of 43 ± 23 L.min^−1^ (Suarez et al. 2002:506–511). Whilst glottic closure is one of the variables needed to build up sufficient pressure for an effective cough, it has been suggested that, in adults without expiratory muscle weakness, glottic closure might not be essential to produce an effective cough if there is a good forced expiratory technique (huff) (Choi et al. 2012:351–354). This could, at least, partly explain why our results show a wide agreement interval, but small bias between PEF and PCF.

Because of the variability in presentation of NMD, some patients may have had difficulty with glottic closure, but still had adequate expiratory muscle strength (lower PCF and higher PEF), whilst others may have weaker expiratory muscles and/or poor mouth closure, but better glottic closure (lower PEF and higher PCF).

The wide limits of agreement suggest that, despite being classified as ‘good’ agreement on ICC, the relationship between PCF and PEF is not close enough to allow for the two variables to be used interchangeably in clinical practice. However, our findings do support the use of PEF to evaluate respiratory muscle function in children with NMD, where other measures are not readily available and/or in the case of impaired glottic function.

The presence of a chest wall deformity significantly affected PEF, but not FVC or PCF, in our study. Colombani (2009) found that children with significant pectus excavatum had below average FEV_1_ at rest, but did not report effect on PEF or PCF (Colombani 2009:58–63). The fact that the presence of a chest wall deformity did not affect PCF or FVC in our cohort is in contrast to a previous report that cough is likely to be impaired in patients with significant chest deformities, owing to an inability to take a sufficiently deep inhalation (Homnick 2007:1296–1305). Severity of chest wall deformity amongst our participants was not always objectively and systematically recorded; if these were mostly mild in nature, this may at least partly explain our findings in this regard. The sample size was too small to conduct sub-analyses amongst different chest deformity types.

This was a single-centre, retrospective study with inherent limitations in terms of data quality and potential bias. Results may not be generalisable to other population groups and contexts. The sample studied was heterogeneous in terms of age, sex, NMD conditions and severity of disease, and the small sample size precluded sub-analyses. There was over-representation of DMD in the study sample, which may have impacted the results, and this should be considered in future studies. It is recommended that further studies, with larger sample sizes, should be conducted to confirm our findings in different settings.

We used PCF cut-off values that have been frequently cited in the literature, but that are largely derived from adult studies. This constitutes a major limitation of this study, and research is recommended to determine appropriate age-related cut-off values of PCF indicative of ineffective cough in young children under 12 years of age.

## Conclusion

We report significant positive, linear correlations between measures of PEF, FVC and PCF, and strong agreement between PEF and PCF. Specific levels of PEF (< 160 L.min^−1^) and FVC (< 1.2 L) were identified as significant predictive factors for poor PCF (< 160 L.min^−1^), whilst PEF < 250 L.min^−1^ was found to be highly predictive of patients at risk of respiratory morbidity during intercurrent infections, corresponding to a PCF < 270 L.min^−1^. PEF and FVC may, therefore, be used as screening measurements to identify patients at risk for pulmonary morbidity related to ineffective cough.
